# Non-nutritive sweeteners and their impacts on the gut microbiome and host physiology

**DOI:** 10.3389/fnut.2022.988144

**Published:** 2022-08-25

**Authors:** Irene L. Richardson, Steven A. Frese

**Affiliations:** Department of Nutrition, University of Nevada, Reno, NV, United States

**Keywords:** non-nutritive artificial sweeteners, saccharin, sucralose, aspartame, gut microbiome, dietary additives

## Abstract

Non-nutritive sweeteners (NNS) are broadly incorporated into foods, especially those representing a growing share of the beverage market. NNS are viewed as a noncaloric and desirable alternative to sugar-based sweeteners and are thought to contribute to reducing overall caloric intake. While these compounds have been studied extensively and have long been considered inert, new research has presented a different view and raises new questions about the effects of NNS on human physiology. Namely, the influence on glucose responses, the gastrointestinal epithelium, and the gut microbiome. As the gut microbiome is now recognized as a major mediator of human health and perturbations to this community are generally associated with negative health trajectories or overt disease, interactions between NNS and the gut microbiome are of increasing interest to clinicians and researchers. Several NNS compounds are now hypothesized to affect human physiology by modulating the gut microbiome, though the mechanism for this action remains unclear. The purpose of this review is to discuss the history and current knowledge of NNS, their reported utility and effects on host physiology and the gut microbiome, and describes a model for investigating the underlying mechanism behind reported effects of NNS on the gut microbiome.

## Introduction

The increased abundance of processed foods among the diets of industrialized nations has led to the overconsumption of non-essential nutrients, such as added or free sugars. Processed food formulations often prioritize consumer perceptions of organoleptic properties of a product, leading many of these goods to contain high amounts of salt and/or sugar. Consequently, overconsumption of added sugar and salt has become a global concern (1–3). To address this concern regarding the consumption of common sweeteners (e.g., sucrose, glucose, natural sugar syrups, and high fructose corn syrup) and their known adverse health effects, food and beverage products now often use non-nutritive Sweeteners (NNS) as replacements for sugar sweeteners.

The use of NNS as sweetening agents and food additives are relatively novel to the human diet and provide a broad range of relative sweetness (4, 5). Overall NNS consumption has almost doubled since the approval of saccharin, the first NNS, however the consensus of safety and efficacy is still debated (6, 7) and NNS remain a controversial topic in food regulatory frameworks worldwide (4, 8–10). Still, their use is widespread as NNS provide a tractable approach to reduce caloric intake, sugar content, and cost (6, 11, 12). The U.S. Food and Drug Administration (FDA), in addition to several international food safety organizations, have assessed numerous NNS as safe for human consumption with no causal relationship between cancer or other health-related issues if consumed within the Acceptable Daily Intake (ADI) (13–15). However, in contrast to the potential benefits of replacing sugar sweeteners with NNS, recent work has implicated the consumption of NNS as being associated with impacts on human physiological responses such as glucose intolerance, as well as cardiovascular disease (16–19).

The gut microbiome has been identified as a major mediator of several physiological processes and communication pathways (e.g., the gut-brain axis) and has been implicated in the modification of xenobiotics (e.g., pharmaceutical drugs) (20–22), and there is now evidence that a similar fate may exist for NNS in the gut (23, 24). Several studies have sought to identify the associations of NNS intake with impacts on human physiology and view the gut microbiome as a mediator of potential effects of NNS on the host (25–27), which raises new questions regarding the safety profile of NNS and whether their interactions with the host and their microbiome are fully understood.

Overall, while the health risks associated with obesity and excess sugar consumption are well known (28–30), there is limited understanding as to whether or how these NNS affect human physiology, whether they may act directly on the host to do so, or if they act indirectly *via* modulation of the gut microbiome. This review will discuss and summarize the current literature regarding NNS and their chemistry, evidence of physiological impacts on the host, and their potential impact of NNS on the gut microbiome.

## The history of NNS, their chemistry, and use

Non-nutritive sweeteners are perceived as a safe and affordable alternative to sugar sweetened beverages (SSBs), particularly in overweight and obese individuals with the goal of limiting caloric intake as well as improving weight management (31–34). Therefore, the prevalence of non-nutritive sweeteners in common diets continues to grow (9, 35). Currently, the US FDA has approved six NNS for use as food additives in the US. These include acesulfame K, advantame, aspartame, sucralose, neotame, and saccharin (Table 1). In addition to two naturally derived zero calorie sweeteners that are Generally Recognized as Safe (GRAS) by the FDA; stevioside and rebaudioside A from the extracts of the stevia plant (*Stevia rebaudiana*), and monkfruit extract (*Siraitia grosernorii*) (42, 43). Though these compounds share an effect of perceived sweetness, their chemical composition and the intensity of their perceived sweetness differs significantly.

**Table 1:** Characteristics of non-nutritive sweeteners approved for use.

**Sweetener**	**FDA approval year**	**Relative sweetness**	**US ADI (mg/kg bw/d)**	**Sweetener servings equivalent to ADI**	**Chemical structure**	**Biologic effects**
Acesulfame K	1988	200 ×	15	23	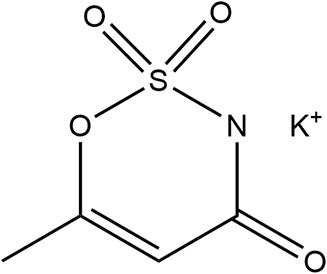	• Not metabolized, rapidly absorbed, excreted intact *via* urine primarily, distributed but no accumulation in tissue (10) • Transfer across placenta, detected in fetal tissue, also detected within breastmilk (10) • Human studies report no effect on PYY or GLP-1 (36)
Advantame	2014	20,000 ×	32.8	4,920	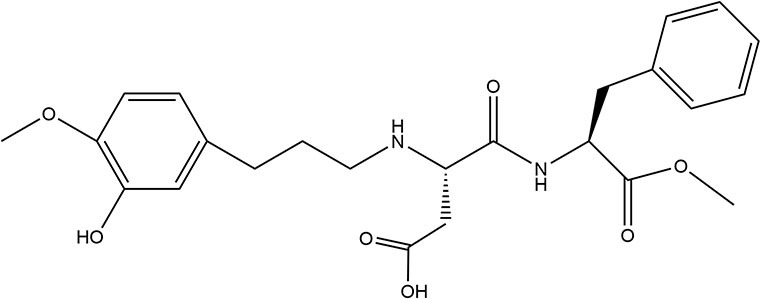	• Promptly hydrolyzed in GIT. Small percentage absorbed (~4–23%) (37) • Primarily (~90%) excreted in feces, remainder expelled in urine (37)
Aspartame	1981	200 ×	50	75	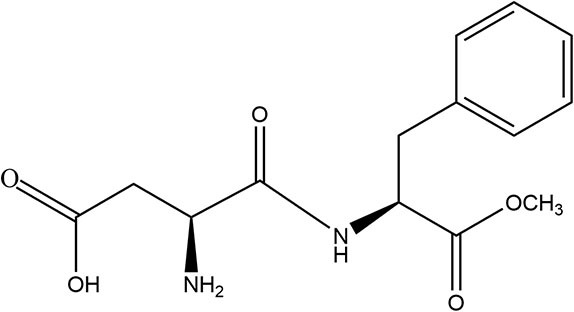	• Hydrolyzed in GIT to three main components aspartic acid, phenylalanine, and methanol (10) • Metabolized in lumen and mucosal cells, absorbed into the bloodstream; Further catabolized into formic acid within humans *via* urine (10) • Human studies report no effect on PYY or GLP-1 (36)
Neotame	2002	7,000–13,000 ×	0.3	23	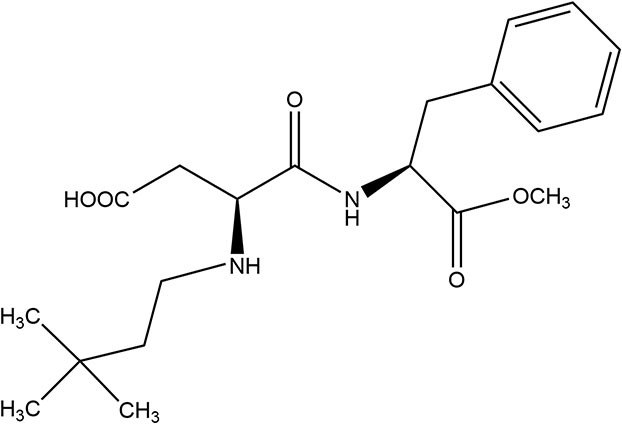	• 50% unabsorbed and excreted in feces, remainder hydrolyzed to methanol (metabolized) and dimethylbutylaspartylphenylalanine (DMB-Asp-Phe) excreted *via* urine (38)
Saccharin	1977	300 ×	15	45	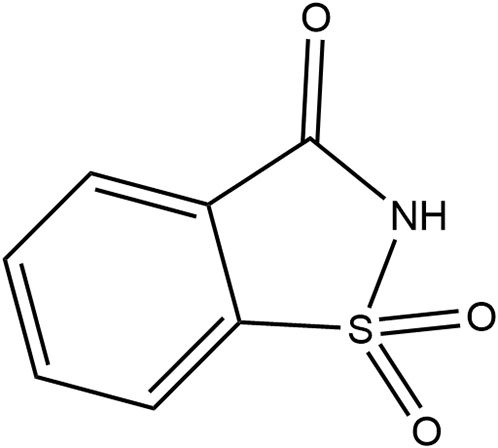	• Not metabolized (~85%−95%) (10) • Absorbed and excreted unchanged in urine and remaining *via* feces (10) • Produces no metabolites (10) • Transfers across placenta and detected in fetal tissue but does not accumulate (10) • Detected in breastmilk (39)
Sucralose	1998	600 ×	5	23	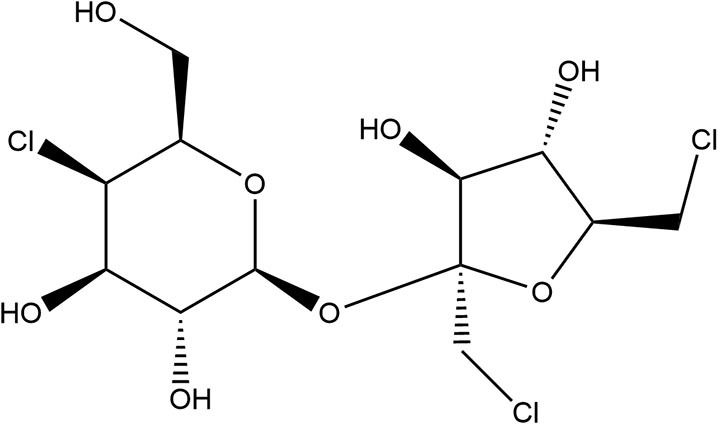	• Majority not absorbed (~85%) and eliminated unchanged in feces primarily (40) • Poorly absorbed (~15%), non-catabolized, readily excreted and no significant effects to GIT (10) • Detected in breastmilk (39) • Several human studies report varying doses produce no effect on PYY, GLP-1, or GIP (36)
Steviol glycosides^a^	2008^b^	200–300 ×	4	9	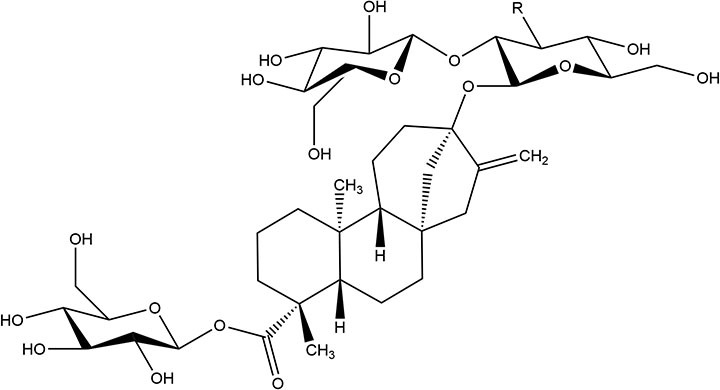	• Of the two compounds (stevioside and Rebaudioside A) no absorption is observed (10) • Compounds firstly hydrolyzed in colon to steviol within ~24 h of ingestion *via* gut microbiome (41) • Absorbed and converted to steviol glucoronide and excreted in urine (5)

ADI, acceptable daily intake; bw, body weight; d, day; PYY, peptide YY.

^a^Stevioside and rebaudioside A differ at the R group; which is either OH (stevioside) or glucose (rebaudioside A).

^b^Characterized as Generally Recognized as Safe (GRAS).

Despite differences in chemical composition, the consensus of safety and regulatory approvals for NNS has led dietary recommendations and health organizations to encourage their use and the suggested beneficial outcomes – primarily as sugar substitutes with little to no caloric cost (44–46). The Academy of Nutrition and Dietetics (AND) has previously reviewed the techniques and evidence as favorable for use in adults with Type 1 and 2 Diabetes (47), if amounts of consumed NNS do not exceed that of the FDA proposed ADI (48). In addition, the AND supports the use of NNS as a strategy for various diet/health concerns including the limiting of carbohydrate and energy intake as well as blood glucose and/or weight management (49).

## Effects of sweeteners on host physiology

Non-nutritive sweeteners carry the advantage over typical sweeteners due to their presumed zero-to-negligible caloric load, as well as producing no direct glycemic effect (48). Despite their extensive usage, the supposed benefits have yet to be established, specifically with reducing body weight. The effects of NNS consumption in relation to body weight management have been largely divided over the main findings and randomized controlled trials in humans are limited (50). Several observational studies have reported weight gain (17, 51, 52), conflicting reports of weight loss (34, 53, 54), or negligible effects on weight (50, 55, 56). A key drawback to many of these studies is determining directionality of the interactions as well as accurate estimates of NNS intake, as these observational studies do not demonstrate causality (44, 57).

A recent meta-analysis of 15 randomized control trials (RCTs) does suggest that there is a modest effect of weight loss in participants who substituted NNS for regular calorie foods (32), which indicates that NNS could be a useful tool to strengthen compliance of weight loss or weight management plans (6). However, the data of the nine reviewed prospective cohort studies suggested a small positive association between NNS intake and body mass index (BMI) whereas evidence from short term observational studies generally have found incomplete energy compensation when NNS was used as a substitution (32). Critically, NNS appear to pose no benefit for weight loss or minimizing weight gain without a restriction of energy intake (6, 58) and are effective only if used as a replacement to caloric sweeteners while also maintaining a caloric deficit (58). Thus, if NNS are used as a substitute to higher calorie alternatives, they do have the potential to aid in weight management (6, 54, 59), though there is no influence of NNS on the hormone incretin in relation to blood glucose, appetite, or weight gain (33). As a result of these varying results the effects of NNS on body weight management, the American Heart Association and American Diabetes Association have both concluded there to be insufficient information to say whether using NNS has the desired impact to reduce body weight (44).

Similar conflicts in the literature have been observed in relation to the influence of NNS on feeding behavior and metabolism (8). Previous work considered these sweeteners predominantly inert in relation to effects on glucose homeostasis because they do not evoke a post-prandial response seen with caloric sweeteners (10, 60). However, NNS are thought to modify energy balance and metabolic functions by means of both central and peripheral mechanisms (61). A strong current theory regarding the physiological interactions is that NNS may impede learned responses regarding glucose control and energy balance (61–65). The cephalic response is considered an innate and learned physiological response to the thought and anticipation of food entering and being digested within the gastrointestinal tract. The major end result is the stimulation of the vagus nerve thereby producing a cascade of actions within the peripheral nervous system (including increased salivation, gastric acid secretion, as well as exocrine and endocrine pancreatic secretions) (66). With continuous exposure to certain foods, the body quickly learns how to respond to an influx of nutrients and for determining satiation. Therefore in the case of sweet compounds entering the oral cavity, like sucrose, a conditioned response is produced looking to predict the caloric uptake as well as compensate with downstream effects to the gut hormones insulin and glucagon-like-peptide-1 (GLP-1) (67). Recent findings report both sucrose and sucralose produce similar stimulations of primary taste pathways (68). Yet, sucrose produces a stronger response and consequently initiates a dopaminergic effect (68) that can be distinguished by the brain, even though the conscious mind could differentiate between the compounds (68). Additionally, saccharin has also been reported to increase insulin levels *via* the cephalic phase in healthy adult humans (69) and while taste receptors can predict caloric consequences, saccharin has been shown to interfere with this ability in foods that tasted sweet (62) and other NNS have been shown to induce insulin release as well (67, 69).

The physiological responses to NNS have been further explored in other work. When comparing the effects of sucralose compared to water consumption in obese, insulin sensitive participants who were atypical consumers of NNS, researchers found sucralose increased plasma glucose and insulin levels, as well as an increase in insulin secretion and decrease in clearance compared to the controls who consumed water. However, there were no differences in several other factors of glycemic response including GLP-1, beta cell sensitivity and glucose dependent insulinotropic peptide (GIP) (70). These findings raise the question of whether non-nutritive sweeteners produce significant effects in obese, insulin sensitive populations for whom glucose levels must be tightly regulated. Finally, while NNS have been shown to bind to sweet-taste receptors and induce GLP-1 release (related to glucose homeostasis) in rodent models and other studies (36, 71), this has not been reproduced with human subjects (71).

The widespread use of NNS has been contingent on the negligible caloric cost as well as no influence on post-prandial responses to appetite and energy, which is essential for both diabetic patients and overweight/obese individuals in search of added sugar replacements. To date, the current findings provide mixed results on the effect of weight management, as well as the effects of NNS on insulin, glucose intolerance, and GLP-1 responses. However, studies examining humans for these responses have not stratified participants by gut microbiome composition, which may explain some of the variability of responses among individuals.

## Evidence for NNS and gut microbiome interactions

The gastrointestinal tract (GIT) provides for the breakdown of most dietary components and enables efficient uptake of nutrients to meet the nutritional needs of the host (72). In contrast, the gut microbiome is a community of microbes which accesses dietary components during and after digestion and absorption and contributes to the fermentation of dietary components such as fiber (72). In doing so, these microbes facilitate the capture of energy from dietary components which the host is unable to access and facilitates the production of additional nutrients (e.g., vitamins) consequently accessible by the host (72). The influence of this community on host development, nutrition, and health is now being understood (72, 73), as more examples of ancient associations between humans and specific gut microbes are identified (74, 75). These ancient associations are significant relationships between humans and our gut microbes as these microbes were recruited and maintained over millennia to perform key functions in the gut (76).

One of the reasons that vertebrates have recruited these gut microbes is that they collectively represent a significant expansion of the genome in terms of enzymatic and metabolic potential by orders of magnitude (77), facilitating the consumption of diets that would otherwise be toxic (78) or completely indigestible (72). Recent work has also identified strong evidence for the impact of this community on the bioavailability and breakdown of xenobiotic compounds (79), and there has been significant interest in understanding how food additives interact with the gut microbiome (80). While some examples have been identified among food additives such as trehalose, whose introduction into the food supply spawned the emergence of pathogenic and trehalose-consuming *Clostridioides difficile* (81), there are few comparable studies examining the impact of other food additives on the gut microbiome.

As NNS are among the most common food additives, whose use in food is relatively recent (82), there has been significant interest in understanding the potential of these compounds to alter gut microbiomes (25, 61, 83, 84). Complicating this effort is the relatively limited, but growing, understanding of the specific enzymatic functions found within the human gut (77) and the diversity of NNS chemical structures (Table 1). Given this challenge, some researchers have tested the impacts of selected NNS on individual members of the gut microbiome, but this has been limited primarily to *Escherichia coli* (85–90).

As the rapid advance of sequencing technologies and analytic software has progressed and costs to generate and analyze the gut microbiome has diminished, there has been an explosion of interest in understanding the gut microbiome and its interactions with the host. There has been some consistency across studies examining gut microbiome responses to the introduction of NNS in rodent models. These studies have spanned the NNS commonly used in foods; saccharin (25, 91), Acesulfame K (92), sucralose (27, 93), rebaudioside A (94, 95), and aspartame (26).

Collectively, studies investigating the impact of NNS on the gut microbiome conclude that while the community may be altered in response to NNS exposure, differences are observed across studies (Table 2), which complicates specific interpretation and direct comparisons while also raising questions as to a potential mechanism of action behind these responses. These findings collectively represent a body of evidence supporting the potential for NNS to alter the gut microbiome, though not all studies have come to this conclusion (104). Notably, these findings have been performed across a variety of murine models providing consistent evidence for the impact of NNS on the gut microbiome, though not all studies show consistent specific changes within the gut microbiome (Table 2). One of the more consistent findings among these studies, however, has been a depletion of *Akkermansia muciniphilia* when exposed to NNS (saccharin, Acesulfame K, and sucralose) in both adult and infant mice (25, 98). When considering the reported effects of NNS on human health parameters like glucose homeostasis (16, 25), several studies in humans and mouse models report that the depletion of *Akkermansia* is associated with increased glucose intolerance (116–118) and mechanistic experiments have identified the secretion of a protein, P9, by *Akkermansia* that induces GLP1 secretion and improves glucose homeostasis in mouse models of obesity and diabetes (119–121). If *Akkermansia* is indeed depleted by NNS consumption, then given the variable distribution of *Akkermansia* among humans (122), this may explain some of the variability with respect to impacts of NNS on glucose tolerance. Further, a small human saccharin challenge study reported that the gut microbiome composition differentiated “responders,” who developed impaired glucose tolerance after consuming the maximum ADI of saccharin for 7 days, and “non-responders” who did not develop insulin resistance (25), which suggests that inter-individual variability in gut microbiome composition may mediate the effects of NNS on host glucose responses. Though this was a small study (*N* = 7), fecal samples from “responders” post-saccharin consumption could recapitulate the glucose intolerance phenotype when transplanted to germ free mice, while fecal samples from the same individuals pre-saccharin consumption did not produce the same effect (25).

**Table 2:** Summary of changes to the gut microbiome in response to NNS.

**Sweetener and ADI**	**Reference**	**Amount used and length of exposure**	**Study type/model**	**Reported effects**
**Acesulfame – K (Ace K)** 15 mg/kg BW/day	(96)	3% Ace K	*In vitro*	• No significant effects
	(97)	1.7–33.2 mg/kg BW/day	Human	• No significant effects
	(93)	15 mg/kg BW/day 8 weeks	Male mice	• No significant effects
	(92)	37.5 mg/kg BW/day 4 weeks	Mice	• Increased *Bacteroides, Anaerostipes*, and *Sutterella* within male rats • Decreased *Lactobacillus* and *Clostridium* within female rats
	(88)		*In vitro E. coli* K-12	• Inhibit *Escherichia coli* HB101 and K-12
	(98)	ADI1x: 0.25 mg AceK + Sucralose (dams only) ADI2x: 0.5 mg AceK + Sucralose (dams only) 6 weeks	Pregnant dams and offspring	• Doubled *Firmicutes* • Diminished *Akkermansia muciniphila*
	(85)	0–6 mg/ml 5 h incubation	*In vitro E. coli* K-12	• Stimulated growth of *E. coli*
	(99)	150mg/kg BW/day 8 weeks	Male mice	• Decreased *Clostridiaceae, Lachnospiraceae, and Ruminococcaceae*
**Aspartame** 50 mg/kg BW/day	(26)	Concurrent with high fat/sucrose diet 5–7 mg/kg BW/day 8 weeks	Rat	• Increase *Enterobacteriaceae* and *Clostridium leptum* within normal chow diet • Increase *Roseburia spp*. with high fat diet
	(100)	135 or 400 mg Single dose	Humans (diabetic)	• No significant effects
	(101)	Concurrent with high fat/sucrose diet 5–7 mg/kg BW/day 18 weeks	Pregnant dams and offspring	• Increase *Porphyromonadaceae*
	(85)	0–6 mg/ml 5 h incubation	*In vitro E. coli* K-12	• Inhibit growth of *E. coli* K-12
	(102)	40mg/kg BW/day (dams only) 6 weeks	Obese pregnant dams and offspring	• Reduced *Limosilactobacillus reuteri* and *Ligilactobacillus murinus*
**Saccharin** 15 mg/kg BW/day	(91)	5 mg/kg BW/day 6 months	Male mice	• At 3 months: Increase *Sporasarcina, Jeotgalicoccus, Akkermansia, Oscillopspira, Corynebacterium*; Decrease *Anaerostipes, Ruminococcus* • At 6 months: Increase *Corynebacterium, Roseburia, Turicibacter*; Decrease *Ruminococcus, Adlercreutzia, Dorea*
Commercial saccharin was used, containing glucose (95%)	(25)	Mice: 5 mg/kg BW/day 5 weeks	Mice	• Mice: Increase *Bacteroides, Clostridiales;* Decrease *Lactobacillus reuteri;* Overrepresented *Bacteroides vulgatis* and Underrepresented *Akkermansia muciniphila* • Human: Increase *Bacteroides fragilis* and *Weissella cibaria*; Decrease *Candidatus Arthromitus*
		Human: 5mg/kg BW/day 1 week	Human	
	(88)	Concurrent with high fat diet 5 mg/kg BW/day 10 weeks	Mice	• Decrease *Tenericutes* • Increase *Proteobacteria* and *Actinobacteria* • Increase Firmicute/Bacteroides ratio • Increase *Akkermansia*
	(103)	0.1 mg/ml 5 weeks	*In vitro* /Mice	• Inhibited *Staphylococcus aureus* (Firmicute), *Klebsiella pneumonia* and *Pseudomonas aeruginosa* (both Proteobacteria)
	(104)	250 mg/kg BW/day (mice) 10 weeks 400 mg/day 2 weeks	Mice Human (Randomized, double-blind, placebo controlled trial)	• No significant effects
	(105)	1.5 mM 4 weeks	Female guinea pig	• Increased *Firmicutes* and *Lactobacillaceae-Lactobacillus*
	(106)	2.5% sodium saccharin Incorporated in feed	Rat	• Inhibited 3 strains of *Lactobacillus* and *E. coli*
	(107)	0.066% (w/v), with or without ethanol (10%) 4 weeks	Mice	• Increased *Eubacteria* in the pregnant group that received ethanol and saccharin • Reduced *Clostridium* population
	(88)		*In vitro*	• Inhibit *E. coli* HB101 and K-12
**Sucralose** 5 mg/kg BW/day	(93)	1.5 mg/kg BW/day 8 weeks	Mice	• Decreased of *Clostridium cluster XIVa*
Commercial sucralose (1.10%), glucose (1.08%), moisture (4.23%), and maltodextrin (93.59%)	(108)	Dosing range (100–1000 mg/kg BW/day) 12 weeks	Rat	• Decreased total anaerobes and aerobic bacteria • Decreased *Lactobacilli, Bifidobacteria, Clostridia*, and *Bacteroirdes*
	(27)	5 mg/kg BW/day 6 months	Male mice	• Increased *Ruminococcus*; Decrease *Lachnospiraceae, Dehalobacteriaceae, Anaerostipes, Staphylococcus, Peptostreptococcaceae, Bacilles* at 3 months • Increase *Akkermansia, Turicibacter, Roseburia, Clostridiaceae, Christensenellaceae*; Decrease *Streptococcus, Lachnospiraceae, Dehalobacteriaceae, Erysipelotrichaceae* at 6 months
	(109)	3.3 mg/kg BW/day (normal chow) 1.5 mg/kg BW/day (high fat diet) 8 weeks	Mice	• Increase *Firmicutes* (normal and high fat diet) • Increase *Bifidobacterium* (normal diet)
	(88)		In vitro	• Inhibit *E. coli* HB101
	(110)	3.5 mg/ml 6 weeks	Mice (induced Crohn's Disease model)	• Increased Proteobacteria
	(98)	ADI1x: 0.1 mg + Ace K (dams only) ADI2x: 0.2 mg + Ace K (dams only) 6 weeks	Pregnant dams and offspring (mouse)	• Increased *Firmicutes*
	(85)	0–6 mg/ml 5 h incubation	*In vitro E. coli* K-12	• No significant effects
	(111)	Concurrent with high fat diet 1.5% water solution 4 months	Male Rat	• increase in three *Bacteroides* species, *B. fragilis*
	(112)	0.1 mg/ml (dams only) 3 weeks	Pregnant dams and offspring	• Increased *Akkermansia, Blautia, Corynebacterium*, and *Robinsoneilla* • Diminished *Alistipes, Barnesiella, Paraprevotella, Saccharibacteria incertae sedis*, and *Streptococcus*
**Steviol glycosides** 4 mg/kg BW/day	(101)	2–3 mg/kg BW/day; 9 weeks	Rats	• *Decrease Bifidobacteriaceae* • *Increase Bacteroides goldsteinii and Bacteroides thetaiotaomicron*
	(113)	2–3 mg/kg BW/day 18 weeks	Obese dams and offspring	• *Decrease Bifidobacteriaceae* • *Increase Bacteroides goldsteinii and Bacteroides thetaiotaomicron*
	(95)	5 mg/kg BW/day Concurrent with high fat diet 10 weeks	Mice	• Increase *Firmicutes/Bacteroides* ratio • Increase *Proteobacteria* and *Actinobacteria*
	(114)	24 h	*In vitro* (human fecal samples)	• *Bacteroides* hydrolyze to steviol and rebaudioside A most efficiently
	(90)	95% (w/w) stevioside 97% rebaudioside A 24 h	*In vitro Limosilactobacillus reuteri*	• Inhibit *L. reuteri* growth
**Neotame** 0.3 mg/kg BW/day	(115)	0.75 mg/kg BW/day 4 weeks	Mice	• Decreased *Firmicutes* • Increased Bacteroidetes

BW, body weight; ADI1x, the recommended ADI; ADI2x, twice the recommended ADI; w/v, weight per volume; w/w, weight per weight; N/A, not applicable.

Other work has sought to examine the inter-generational impact of NNS on offspring, as NNS can be detected in milk (39). Low dose aspartame (5–7 mg/kg) and stevia (2–3 mg/kg) was associated with alterations to adiposity, insulin sensitivity, glucose tolerance, as well as the mesolimbic reward pathway in pregnant rats (101). While there were minimal differences observed within the fecal microbiota of these animals, relative to the control group, *Clostridium leptum* is noted to be more abundant within both groups of dams and offspring receiving sweetener supplementation, but *C. leptum* was not carried over to the offspring of dams receiving water alone (101). There was also an enrichment of the family *Porphyromonadaceae* within the offspring of rats fed aspartame or stevia. Importantly, the transplant of fecal samples from the offspring of sweetener supplemented dams to germ free mice produced similar physiological effects observed among the NNS-supplemented animals including increased body weight, percent fat mass, and a trend toward reduced glucose tolerance (101). These ex-germ-free mice also displayed an increased abundance of *Porphyromonadaceae*, comparable to the offspring exposed to aspartame and stevia within the mother's diet. Importantly, this work highlights the impact of these microbiome alterations on host glucose responses and demonstrated that these changes, which resulted from direct exposure to NNS, could impair health in germ-free animals receiving this microbiome composition without the NNS itself (101).

In humans, the effects of artificially sweetened beverage (ASB) consumption by pregnant women has been studied to examine the effects of NNS on infants, particularly the gut microbial composition and the associated function within the initial year of life (123). In a prospective study, infants (*N* = 100) from 3 to 12 months of age were studied. Half of the study population were infants born to mothers consuming ASBs during pregnancy and half were born to mothers who did not. Infants born to mothers who consumed ASBs were found to have a higher BMI, compared to children of mothers who did not consume ASBs during pregnancy (123). Infant fecal samples were also used to compare the gut microbiome composition of these infants, which identified associations between maternal ASB consumption and beta diversity, as well as a depletion of *Bacteroides sp*. and enrichment of *Provotella copri*. The authors reported secondary effects of increased BMI to be associated with higher levels of the urine metabolites spermidine and succinate within exposed infants (123) and elevated succinate circulation has been previously associated with obesity and impaired glucose metabolism (124).

Collectively, the outcomes from these studies investigating the impact of NNS on the gut microbiome suggest that not only is the gut microbiome affected by the consumption of NNS, but that these impacts on the gut microbiome have physiological consequences for the host (25, 88, 98), and that these consequences may be transmitted vertically, from mother to offspring. As NNS can be detected in milk (39), these findings raise questions as to whether these impacts on the gut microbiome of offspring are related to prenatal impacts on the maternal gut microbiome or maternal provision of NNS *via* milk (39, 125–128), as well as what other confounding lifestyle factors may shape the gut microbiomes of both mothers and their offspring (129).

## Potential mechanisms for interactions between gut microbes and NNS

While regulatory review of each sweetener includes extensive toxicology and safety data (14, 130–132), new research related to the gut microbiome has raised questions as to how these NNS have effects on host physiology. When considering the possible mechanism underlying these results, there are several potential routes by which these findings may be rationalized and supported in future work.

First, there may be interactions between NNS and either known taste receptors or unappreciated receptors with affinity for these compounds found in the gut and linked to the capacity for glucose absorption and homeostasis (133). This possibility would suggest that it is not necessary for NNS to be absorbed to shape host physiological responses and by their regular inclusion in foods that we perceive to be sweet, this alone is sufficient to trigger a physiological response as if these compounds were sugar sweeteners themselves, as cephalic phase insulin release appears to have a contextual component (134). While this is certainly a possibility, the absence of consistent evidence supporting insulin release in response to NNS complicates this possibility (57).

**Figure 1:**
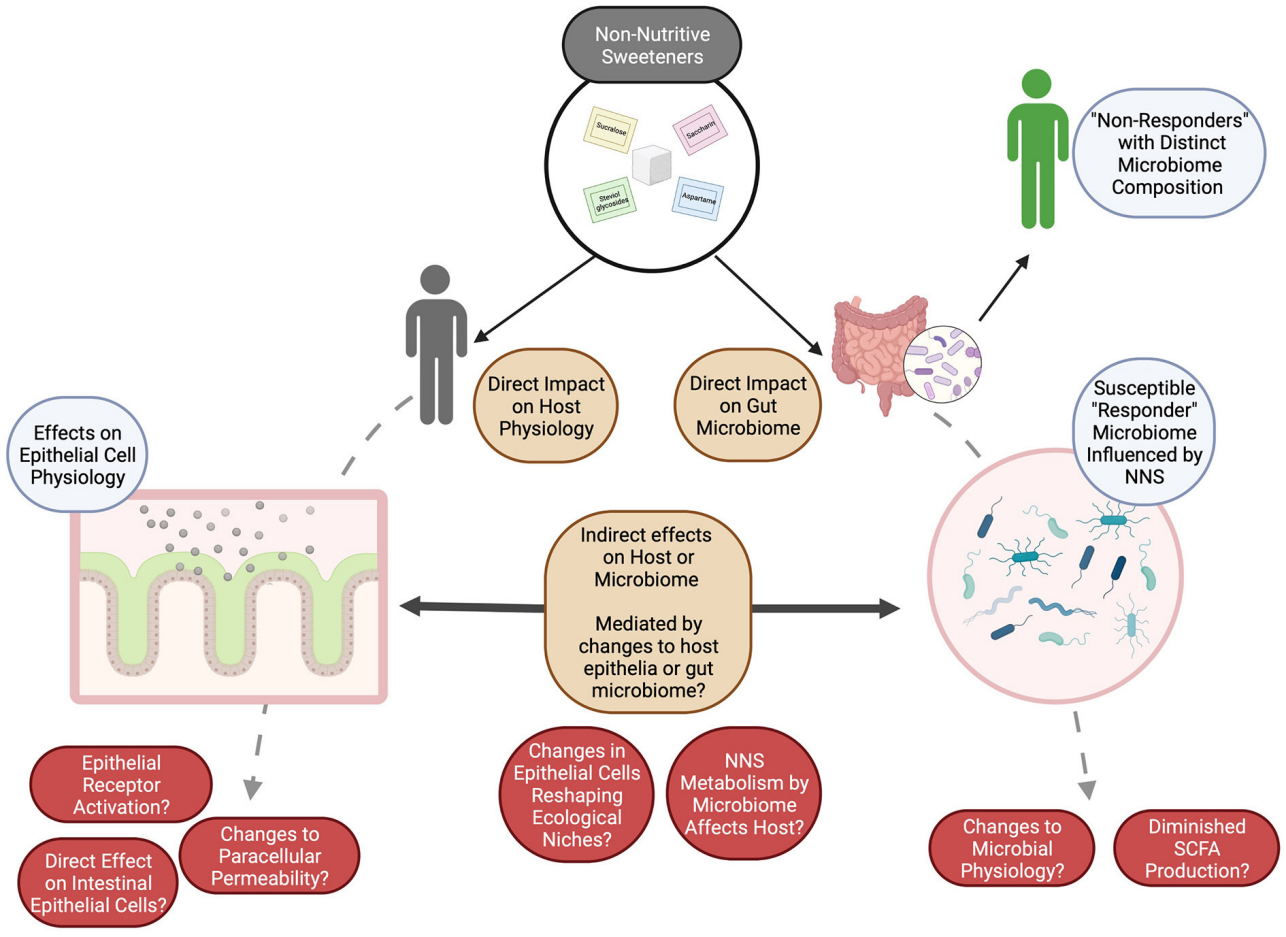
The potential for NNS to shape both the gut microbiome and host responses to these compounds may be either direct or indirect. Many open questions remain (in red) as to how these impacts or interactions may be measured or monitored, but we speculate (brown) that by investigating the impacts of NNS on (1) the host (especially on morphologically distinct epithelial cells), (2) on the gut microbiome, as a whole, and (3) on the interactions between the gut microbiome and the host, the underlying mechanisms can be described and evaluated for their potential to influence human health. Figure created with BioRender. SCFAs, short chain fatty acids.

Alternatively, these compounds may act directly on the gut epithelium to shape gut epithelial processes, like mucin production and gut barrier function (76, 135–137), which typically regulate the gut microbiome and shape its composition and metabolism (135, 138). These compounds may also have acute effects on keystone species within the gut microbiome itself, and major impacts on mucin production or its structure have been associated with the depletion of taxa reliant on mucin glycans, such as *Akkermansia* (120). Further, there is some evidence that several distinct NNS can damage bacterial cell membranes and alter cellular permeability with an “antibiotic-like” effect (102). Conversely, the breakdown products of these compounds, either by the host (8, 65, 136, 139) or the gut microbiome (8, 25, 60, 108), may affect the gut microbiome or the gut epithelium and shift microbial populations as has been reported (Table 2; Figure 1).

Together, the reported effects of NNS on human physiology (e.g., impaired glucose tolerance) can be conceptually differentiated to be (1) directly active on the host to influence physiological responses, (2) act on the host epithelium and indirectly influence the gut microbiome composition to influence host responses, or (3) act directly on the gut microbiome to influence its composition. While there is evidence of the first possibility, studies incorporating the microbiome composition of individuals and/or using microbiome transplantation experiments appears to more strongly support an effect of NNS on the gut microbiome, either directly or indirectly, which then influences host health, as generally reported in terms of glucose intolerance, increased body weight, or metabolic modifications (25, 123).

## Conclusion

In this review, we assess the microbial and associated metabolic effects of non-nutritive sweeteners and recognize controversies/shortcomings of the existing evidence behind these structurally varied compounds and their use. While there is extensive safety evidence behind NNS, there are growing bodies of work which suggest that NNS in high concentrations may exert possible negative health outcomes within certain susceptible populations/individuals. In particular, susceptible populations may ultimately be identified by their gut microbiome composition, rather than obvious clinical features, given findings identifying responder/non-responder differences among individuals in a small saccharin challenge study (25, 82, 140).

The determination of these effects entails critical evaluation of previously reported confounding factors and a more recognized understanding that each NNS carries individual potential to explain unique metabolic or sensory effects observed (141). Utilizing well-designed and appropriately powered studies in humans, in addition to relevant animal or *in vitro* models that reflect the human gut microbiome, are critical to comprehend these reported alterations to microbial populations and evaluate their consequences for human health (97, 142). Additionally, the use of gnotobiotic mice have been recognized as one the most informative model when experimentally evaluating responses of the human gut microbiome to dietary challenges (142).

Finally, despite previously unappreciated impacts of NNS, their value must be considered in the context of their role in limiting caloric intake, as alternatives to sugar-sweetened beverages. The value of NNS to efforts limiting the global health burden of obesity and obesity-related disease may outweigh potentially negative effects on human health. While observational studies have linked NNS consumption with an increased risk of cardiovascular disease (17, 143) and acute impacts on glucose responses have been described (16, 25), it is unclear whether short-term consumption is associated with the same outcomes. Further, if acute effects on glucose responses are reversible, and if NNS are consumed in moderation with concomitant caloric reduction, perhaps these food additives can be useful to reduce the significant health risks associated with obesity, which may outweigh the risks of NNS consumption (144). Further research is clearly needed to characterize and assess the potential for NNS to affect human health and the gut microbiome, as well as supporting mechanistic data to identify how these impacts occur. Future studies examining NNS should especially consider the gut microbiome of the study population, whether in animal models of human studies, to more closely determine the relative value of NNS in limiting obesity.

## Author contributions

ILR and SAF wrote the manuscript. All authors approved the final manuscript.

## Funding

The authors are supported by the University of Nevada, Reno College of Agriculture, Biotechnology, and Natural Resources (CABNR) and the Nevada Experiment Station; the University of Nevada, Reno Department of Nutrition; and the Office of the Vice President for Research and Innovation at the University of Nevada, Reno.

## Conflict of interest

The authors declare that the research was conducted in the absence of any commercial or financial relationships that could be construed as a potential conflict of interest.

## Publisher's note

All claims expressed in this article are solely those of the authors and do not necessarily represent those of their affiliated organizations, or those of the publisher, the editors and the reviewers. Any product that may be evaluated in this article, or claim that may be made by its manufacturer, is not guaranteed or endorsed by the publisher.
